# Multiple biomarker responses (serum biochemistry, oxidative stress, genotoxicity and histopathology) in *Channa punctatus* exposed to heavy metal loaded waste water

**DOI:** 10.1038/s41598-017-01749-6

**Published:** 2017-05-10

**Authors:** Mehjbeen Javed, Md. Irshad Ahmad, Nazura Usmani, Masood Ahmad

**Affiliations:** 1 Aquatic Toxicology Research Laboratory, Department of Zoology, Aligarh Muslim University, Aligarh, 202002 Uttar Pradesh India; 2 Department of Biochemistry, Faculty of Life Sciences, Aligarh Muslim University, Aligarh, 202002 Uttar Pradesh India

## Abstract

Experiments were conducted to investigate the health of fish *Channa punctatus* inhabiting heavy metal-loaded waste water. Heavy metals in the order of Fe > Mn > Zn > Co > Ni > Cu = Cr were present in the waste water. Gills had high metal load followed by liver and then kidney. Albumin, albumin to globulin (A:G) ratio, triglyceride, high density lipoprotein (HDL) and very low density lipoprotein (VLDL) were found to be lower but phospholipid, low density lipoprotein (LDL), total protein, lipid and cholesterol were higher as compared to the reference. Oxidative stress markers such as superoxide dismutase (SOD), catalase (CAT), glutathione S transferase (GST) and lipid peroxidation (LPO) were significantly higher in all tissues, whereas reduced glutathione (GSH) levels were comparatively low. Damage to DNA was observed with significantly higher mean tail length of comets in the exposed fish gill cells (30.9 µm) followed by liver (24.3 µm) and kidney (20.6 µm) as compared to reference fish (5.2, 4.8 and 5.9 µm respectively). Histopathology in gill, liver and kidney also showed marked damage. Integrated biochemical, oxidative stress, genotoxicity and histopathological findings are valuable biomarkers for native fish adaptive patterns, and monitoring of water quality/pollution of freshwater ecosystems.

## Introduction

Heavy metals are widespread in all compartments of environments thereby contaminating water, soil and biota and ultimately affecting human health^1^. They are released from variety of sources including domestic, industrial and agricultural activities gaining entry into the aquatic ecosystem. Heavy metals are persistent and non-biodegradable with the tendency to accumulate and cause deleterious effects in living organisms^2^. They exert toxic effects on survival of fish and other aquatic biota and their overload in tissues may also affect oxidative metabolism such as glycolysis, protein and lipid profile of body^3^. Many of them also impair health through redox cycling by producing reactive oxygen species (ROS) or free radicals which later cause the oxidative stress, DNA damage and lesions in tissues^4^. The production of ROS has been reported to increase particularly due to the exposure to transition metals [Cr, Mn, Fe, Co, Ni, Cu and Zn] because they are redox active chemicals^5^. These free radicals being bind to hyperactive, protein and DNA and thus cause changes in their structures. Therefore, it is essential to remove ROS and/or other free radicals for maintaining physiological balance within the body and to prevent the development of pathological processes^4^. For neutralization of free radicals antioxidant defense system is present in the animal which includes both enzymatic and non-enzymatic systems. Among enzymatic systems the role of superoxide dismutase (SOD), catalase (CAT), glutathione S transferase (GST) is documented whereas reduced glutathione (GSH) is one of the important non-enzymatic antioxidant. If available antioxidants are insufficient to quench all free radicals then chances of DNA damage with concomitant tissue damage become higher. Moreover, during stress the energy requirements in animal body increase and biomolecules like glucose, lipids and protein, also start mobilizing to meet the energy demand depending on the intensity of stress^6^. Current research on alteration in serum biochemistry, oxidative stress, DNA damage, histopathology and their defense by antioxidant system has a more widened scope because of its potential benefit in disease prevention and health promotion^7^. Consequently, an increasing number of responses in animal models under different experimental and ecological conditions are being documented to elucidate mechanisms of action of oxidants or antioxidants^8, 9^.

Previously, many researchers focused attention on the effect of xenobiotics on mammalian models^9–11^. However, effect on aquatic organisms such as fish has not been sufficiently investigated despite their great economic importance. Therefore, the aim of the present study was to investigate the effect of contaminating waste water on the inhabiting fish, *channa punctatus*. The parameters included herein were serum glucose levels, protein and lipid profile; oxidative stress, genotoxicity and histopathology of reference and exposed fish. *C*. *punctatus* has been classified by International Union for Conservation of Nature (IUCN) under red listed species. Moreover, it thrives well in polluted water bodies. Therefore, it was picked to check its health status and to use this fish as bioindicator.

## Results and Discussion

The heavy metal concentrations in water of Panethi reservoir were recorded in the order: Fe (9 mgL^−1^) > Mn (2.5 mgL^−1^) > Zn (0.54 mgL^−1^) > Co (0.26 mgL^−1^) > Ni (0.1 mgL^−1^) > Cu (0.08 mgL^−1^) = Cr (0.08 mgL^−1^) where Cr, Mn, Fe and Ni were found to exceed the recommended guidelines of United Nations Environment Programme Environmental Monitoring System (UNEPGEMS)^12^ and Bureau of Indian standards (no guidelines for Ni; IS 2296: 1992). At reference site (Sumera reservoir) Fe concentration was found to be 0.24 mgL^−1^ and Cu content was 0.1 mgL^−1^. While Cr, Mn, Co, Ni and Zn were below the detection limits. Fe was highly bioavailable and Co was lowest at Panethi site and the order was Fe (97%) > Zn (88.88%) > Mn (77%) > Cu (47.6%) > Cr (28%) > Ni (22.5%) > Co (11%). Whereas at reference site, Fe and Cu available to biota were recorded to be 7.7% and 5.3%. This data is provided as supplementary material in Supplementary Table S1.

Accumulation of heavy metals in gill, liver and kidney of *C*. *punctatus* is given as Supplementary Table S2. In gills of exposed fish, Fe (17619 mg/kg d.w) accumulation was the highest and Ni (31.59 mg/kg d.w) the lowest. In liver also, Fe (1501 mg/kg d.w) was the maximum and Cr (23 mg/kg d.w) the minimum. Similarly, kidney has highest Fe (3554 mg/kg d.w) and lowest Cr (10 mg/kg d.w). In the reference fish Fe accumulation followed by Cu in gills, liver and kidney. In both the exposed and the reference fish, it was observed that gills had a high metal load followed by liver and kidney respectively.

Data on glucose content as well as protein and lipid profile in the serum is given in Table 1. Low glucose content was found in fishes obtained from Panethi reservoir as compared to the reference site. It has been reported by other investigators that under prolonged exposure to heavy metals serum glucose level first elevates and then declines until it attains a depleted level^13, 14^. This could be due to depletion of energy reserves to cope up with stress caused by high accumulation of heavy metals. Low glucose content in chronically exposed fishes observed in polluted waters could also be due to improper gluconeogenesis. Fishes inhabiting at contaminated site showed relatively higher total protein as compared to the reference whereas albumin was low. Lower albumin may be due to liver damage as evidenced in histopathological study. Moreover, a higher serum globulin value but lower A:G ratio was observed. These observations agree well with Gopal *et al*.^15^. In another field study, conducted by our group, significantly higher levels of total protein and globulin but low albumin and A:G ratios were reported in *C*. *punctatus* dwelling in polluted canal with thermal power plant effluents^6^. Increase in globulin content might be due to increased production of vital proteins combating metal toxicity. The protein synthesis could also be needed to meet out the demand for the repair of damaged tissues and heightened immune response. As albumin and globulin are among the most abundant proteins in the animal kingdom^6^. Albumin is produced by liver and globulin by many parts of body^16^. In humans albumin is responsible for approximately 80% of the colloid-osmotic pressure between blood and tissue fluids^17^. In the current study globulin level was comparatively high which may reflect the heightened immunological defense response due to toxicants. A:G ratio is an index used to track changes in the composition of serum or plasma and its normal value lies between 0.8 and 2.0 in case of humans^17^. At the test site, A:G ratio was below 0.8 value which would probably indicates the onset of pathological processes. Albumin, globulin and their ratio (A:G) are used to predict the liver and kidney disorders. Furthermore, increased protein levels would not necessarily reflect the healthy status of fish rather the ratio of albumin to globulin gives the clear picture as can be seen in these chronically exposed fishes. Estimation of total protein alone to check the environmental effect would therefore, be misguiding.

**Table 1:** Serum glucose levels, protein and lipid profile of *Channa punctatus* collected from reference and polluted sites.

Variables	Sumera reservoir	Panethi reservoir	Percent change over reference
Glucose (mg%)	1.69 ± 0.06	1.06 ± 0.08	(−37.27%)
Total protein (mg/ml)	2.49 ± 0.01	3.33 ± 0.33^*^	(+33.73%)
Albumin (mg/ml)	1.56 ± 0.01	0.98 ± 0.03^*^	(−37.17%)
Globulin (mg/ml)	0.93 ± 0.004	2.35 ± 0.3	(+152.68%)
A:G ratio	1.67 ± 0.001	0.42 ± 0.04^**^	(−74.85%)
Total Lipid (mg/dL)	489.01 ± 0.50	533.25 ± 1.6^*^	(+9.04%)
Total Cholesterol (mg/dL)	149.41 ± 0.45	227.78 ± 0.6^**^	(+52.45%)
Phospholipid (mg/dL)	199.06 ± 0.32	256.28 ± 0.5^*^	(+28.74%)
Triglycerides (mg/dL)	142.60 ± 0.23	49 ± 0.5^*^	(−65.49%)
HDL (mg/dL)	62.55 ± 2.65	56.7 ± 0.1^*^	(−9.35%)
LDL (mg/dL)	58.34 ± 1.65	161 ± 0.6^*^	(+176.31%)
VLDL (mg/dL)	28.52 ± 0.45	9.84 ± 0.2^*^	(−65.49%)

Values are given as mean ± SEM, (n = 10); Level of significance established at *p < 0.05 and **p < 0.01; +/− sign indicates high or low over reference.

Lipid is also an important energy source and an essential component of cell membrane (phospholipids and cholesterol). Besides this they also play a significant role as messengers in signal transduction pathways and molecular recognition processes^18^. Hence, any changes in lipid metabolism would signal to impairment of these crucial pathways. In these chronologically exposed fishes, total lipids, total cholesterol and phospholipids levels were comparatively high. Similar observations have also been made by other investigators^19, 20^. Cholesterol, phospholipids, and triglycerides combine to form total lipids; hence elevation in these components (phospholipids and cholesterol) is directly proportional to total lipids. High cholesterol content in the blood/serum could also be due to the transport of lipid from the synthesis site for subsequent utilization either through oxidation or a process of gradual instauration of lipid molecules^20^. Liver dysfunction and disturbance of lipid metabolism also favors elevation in these components. Being an important structural component of cell membrane lipids maintain fluidity, so membrane degeneration could be another possible cause of their elevation. The values for triglycerides and VLDL were low in exposed fish as compared to those in the reference. Corroborating values of triglycerides were reported in *Labeo rohita*^21^. Abalaka^22^ also reported low triglycerides values in *Clarias gariepinus* under wild conditions. It has been reported that constant energy demand leads to mobilization of triglycerides since they serve as lipid depots^18, 23^. A decline in triglycerides could also be correlated to their utilization in membrane biogenesis^18, 23^. Decreased triglyceride may also be due to low feed intake or low absorption due to gut damage or improper synthesis in the liver. VLDL is the triglyceride-rich lipoprotein and its concentration depends on the triglyceride fraction^18, 23^. HDL level was also low in the heavy metal exposed fishes. Low HDL content was also reported in *Oreochromis niloticus* on exposure to heavy metals^24^. It could be due to lipid peroxidation. HDL is also known to help scavenge cholesterol from extra hepatic tissues. Hence, decrease in HDL content could be correlated with increasing cholesterol levels^25^. However, LDL levels were high relative to those in reference fishes. Metwally^24^ also reported a heavy metal induced rise in serum LDL. Kojima *et al*.^26^ attribute it to changes in gene expression of some hepatic enzymes like HMG-CoA reductase (hydroxyl-methylglutaryl- CoA), which would suppress LDL-receptor gene expression.

**Figure 1:**
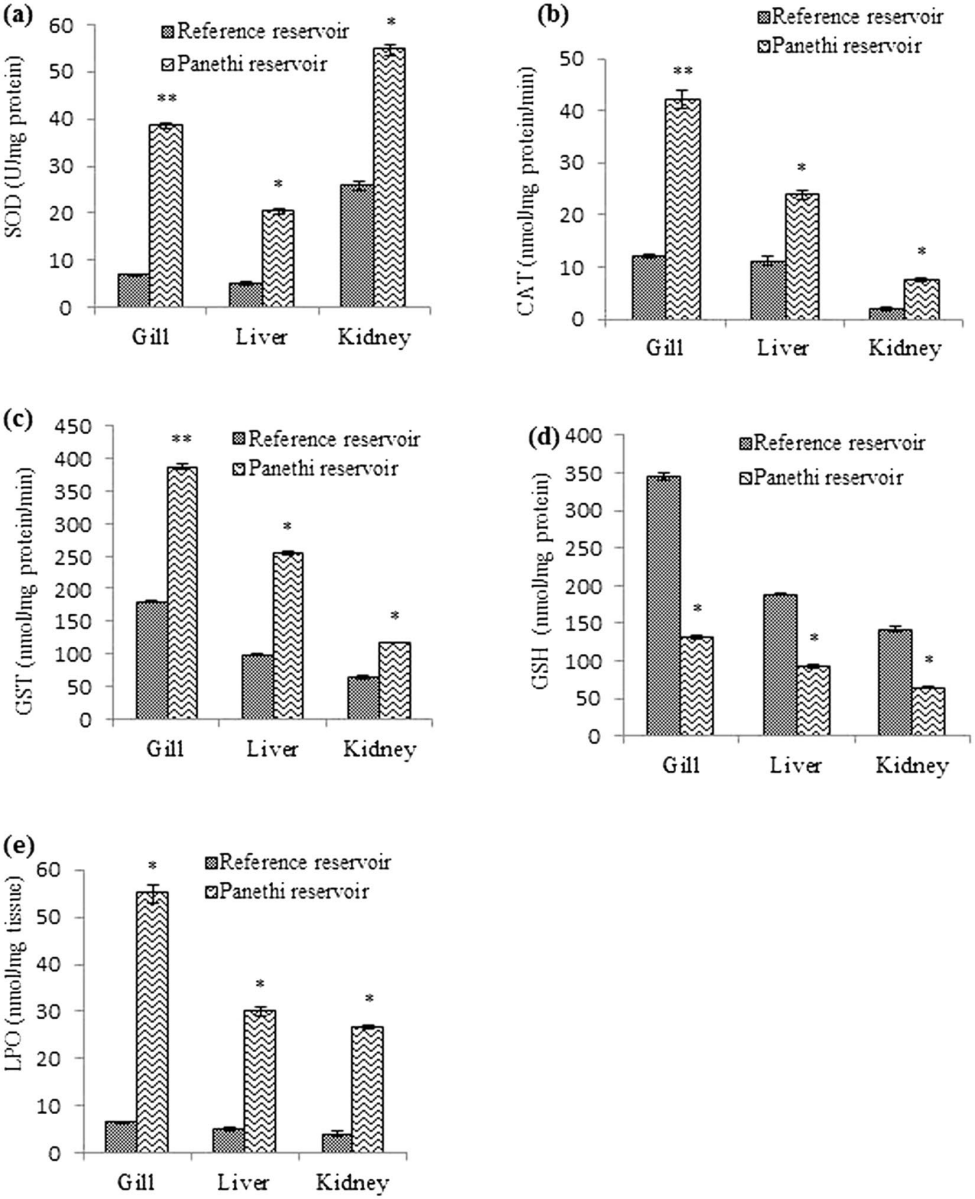
Oxidative stress biomarkers in *C. punctatus* inhabiting in reference and contaminated water. All values are expressed as mean ± SEM, (n = 10); statistically significant at *p < 0.05; **p < 0.01; SOD (superoxide dismutase), CAT (catalase), GST (glutathione S transferase), GSH (reduced glutathione), LPO (lipid peroxidation).

The data of SOD, CAT, GST, GSH and LPO in gill, liver and kidney of *C*. *punctatus* is summarized in Fig. 1. There were significantly higher levels of SOD, CAT, GST but lower GSH levels in the test tissues of fishes chronologically exposed to heavy metals compared to fish from reference site. Activities of SOD (38.5 U/mg protein), CAT (42.2 nmol/mg protein/min), GST (387.3 nmol/mg protein/min) were found to be elevated in gills while GSH level (131.7 nmole/mg protein) was low compared to that in reference fish. Similarly the activities of SOD (20.3 U/mg protein), CAT (23.9 nmol/mg protein/min), GST (254.3 nmol/mg protein/min) were also elevated in the liver while GSH level (93 nmole/mg protein) was lower than in the reference fish. Similar to gill and liver, kidney also showed similar trend. Activities of SOD (54.6 U/mg protein), CAT (7.61 nmol/mg protein/min) and GST (117.6 nmol/mg protein/min) were relatively high in kidney whereas a lower levels of GSH (64.8 nmol/mg protein) were recorded in the fishes of the contaminated than those in fresh water (reference). Similar observations have also been recorded in gill, liver and kidney of other and same species of fishes such as *Carassius auratus*^27^, *B*. *bocagei*^28^ and *C*. *punctatus*^29^. The heavy metals (Cr, Mn, Fe, Co, Ni, Cu and Zn) accumulated in these tissues are all potentially redox active suggesting an imbalance between production of ROS and their neutralization, and bearing fish is said to be under the influence of oxidative stress. This imbalance may lead to damage of tissues and cellular components, which in turn would trigger induction of antioxidant defense mechanisms^30, 31^. Hence, SOD, CAT, GST and GSH can serve as sensitive biomarkers of environmental pollution in aquatic organisms. SOD converts the superoxide radical anion (O^.^^−2·^) to H_2_O_2_, its activity in gill, liver and kidney was elevated compared to the reference fish. Likewise, CAT activity also increases to destroy the H_2_O_2_ which could otherwise penetrate through the biomembranes and may inactivate several enzymes^32^. GST is the phase II biotransformation enzyme which catalyzes conjugation of electrophile to glutathione (GSH). We found an enhanced level of GST activity in fishes inhabiting in effluent contaminated water reservoir. GSH is also capable of scavenging ROS. Contrary to increased activity of GST, GSH levels were found to be low in gill, liver and kidney of exposed fishes. Depletion of GSH could be due to its oxidation to GSSG^31^.

There were significantly (p < 0.05) higher levels of lipid peroxidation (LPO) in gill (55 nmol/mg tissue), liver (30 nmol/mg tissue) and kidney (26.7 nmol/mg tissue) of waste water exposed *C*. *punctatus* clearly indicating the membrane damage. Other investigators have also reported LPO elevations in various varieties of fish such as *Goodea atripinnis*^33^, *B*. *bocagei*^28^, *C*. *punctatus*^29, 34^. Level of LPO mainly depends on the availability of polyunsaturated fatty acids (PUFA) and antioxidant defense. Fishes are considered as the rich source of PUFA. The metals (Cr, Mn, Fe, Co, Ni, Cu and Zn) detected herein are all redox active, accumulated significantly in fish tissues and could contribute to the generation of ROS which are responsible for damaging lipids, proteins and DNA.

**Figure 2:**
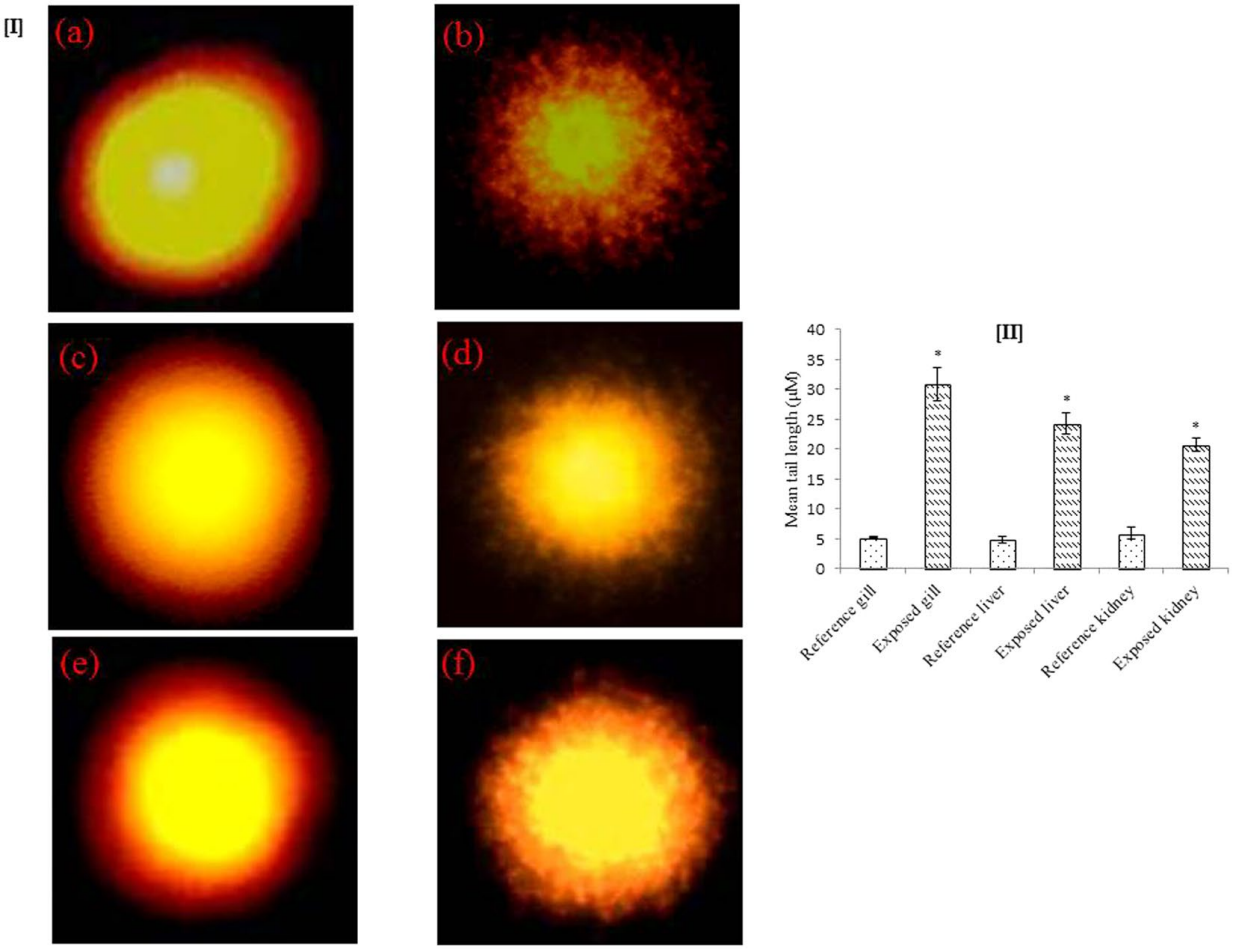
[I]. DNA damage as visualized by comet assay in reference site and panethi reservoir in gill, liver and kidney cells. (**a**, **c** and **e**) are the reference groups and (**b**, **d** and **f**) are the exposed groups of gill, liver and kidney cells of *C. punctatus* respectively. [II]. Mean tail length (µm) of DNA comets in *C. punctatus* in gill, liver and kidney tissues collected from reference and exposed samples. All values are given as mean ± SEM, (n = 10); Level of significance established at *p < 0.05

In every living being, the genome governs cell functioning in response to signals from its environment. Any physical or chemical agent and xenobiotic capable of interfering with these signals may be toxic for cell cycle, growth, division and differentiation^35^. Figure 2 [I] and [II] illustrated DNA damage in gill, liver and kidney of exposed *C*. *punctatus*. A significantly (p < 0.05) higher mean tail length was observed in exposed gill (30.9 µm) as compared to that in the reference (5.2 µm) fish. Similarly, in liver (24.3 µm) and kidney (20.6 µm) of test fish showed higher DNA damage than in liver (4.8 µm) and kidney (5.9 µm) of reference fish. Other investigators have also reported higher DNA damage due to heavy metal exposure (Fe, Cu, Zn, Pb, Cd, Cr, Mn, Co, Ni and Zn) in fishes such as *C*. *punctatus*^34^, *Limanda limanda*^36^, *Leuciscus cephalus*^37^, Ahmad *et al*.^38^ reported that DNA integrity in gills of *Anguilla anguilla* was lost at 1 mM Cr concentrations without any perceptible change at 100 µM. DNA plays a vital role in processes like gene transcription, gene expression, carcinogenesis and mutagenesis^39^. It have been reported that some of these metals form DNA adducts by intercalating or covalently binding with the DNA molecule. If the adducts are misrepaired or not repaired at all before DNA replication, then they may cause gene mutations and initiate carcinogenesis in animals and humans^40^.

**Figure 3:**
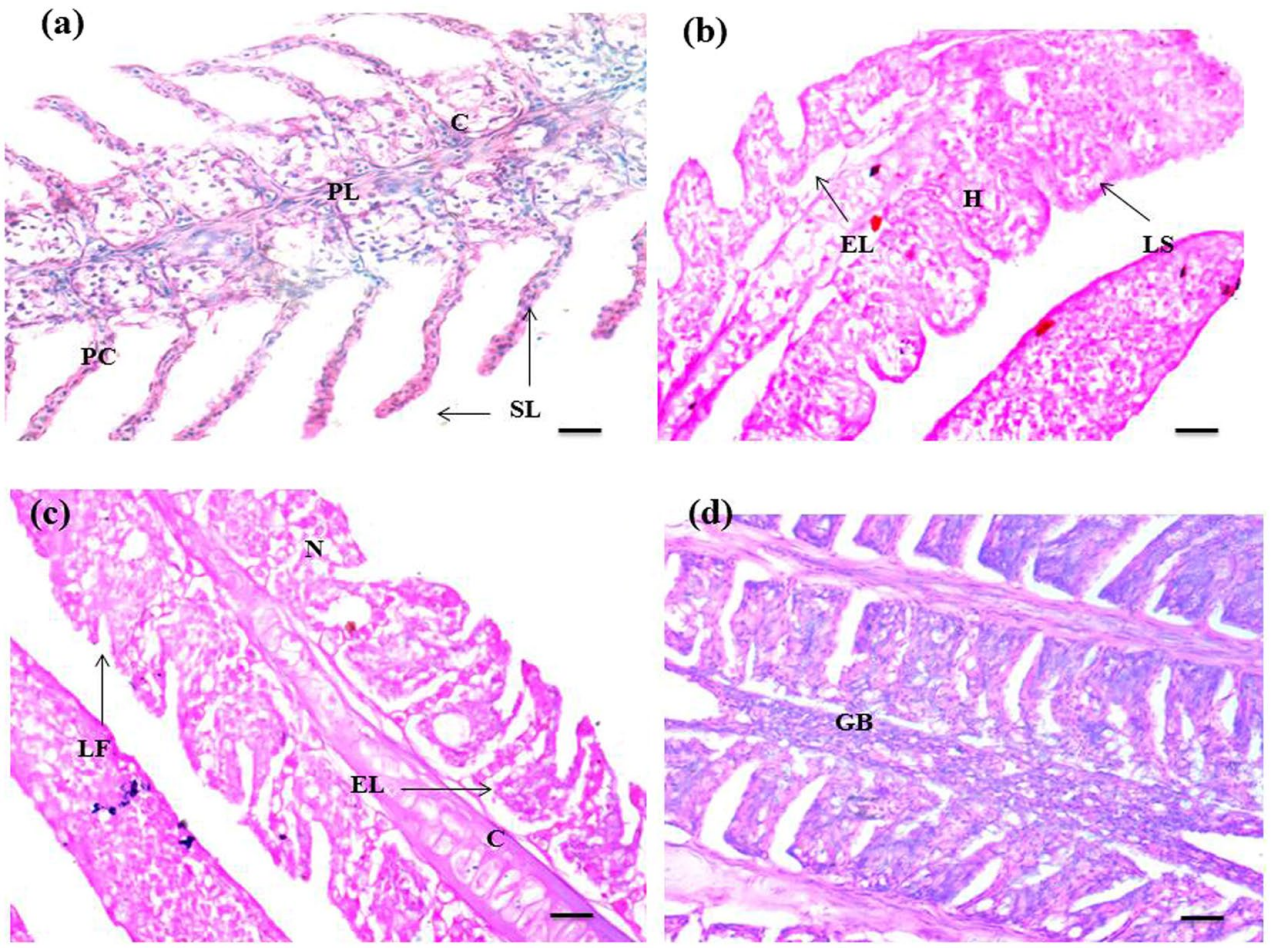
Histopathology of gill of *C. punctatus* inhabiting in reference and contaminated water. (**a**) Gill of reference fish; (**b**, **c** and **d**) Gill of exposed fish; PL (primary lamellae), PC (pillar cell), E (erythrocyte), C (cartilage), SL (secondary lamellae), LF (lamellar fusion), GB (gill bridging), EL (epithelial lifting), HP (hyperplasia), N (necrosis), LS (lamellar swelling). Scale bar = 20 µm.

**Figure 4:**
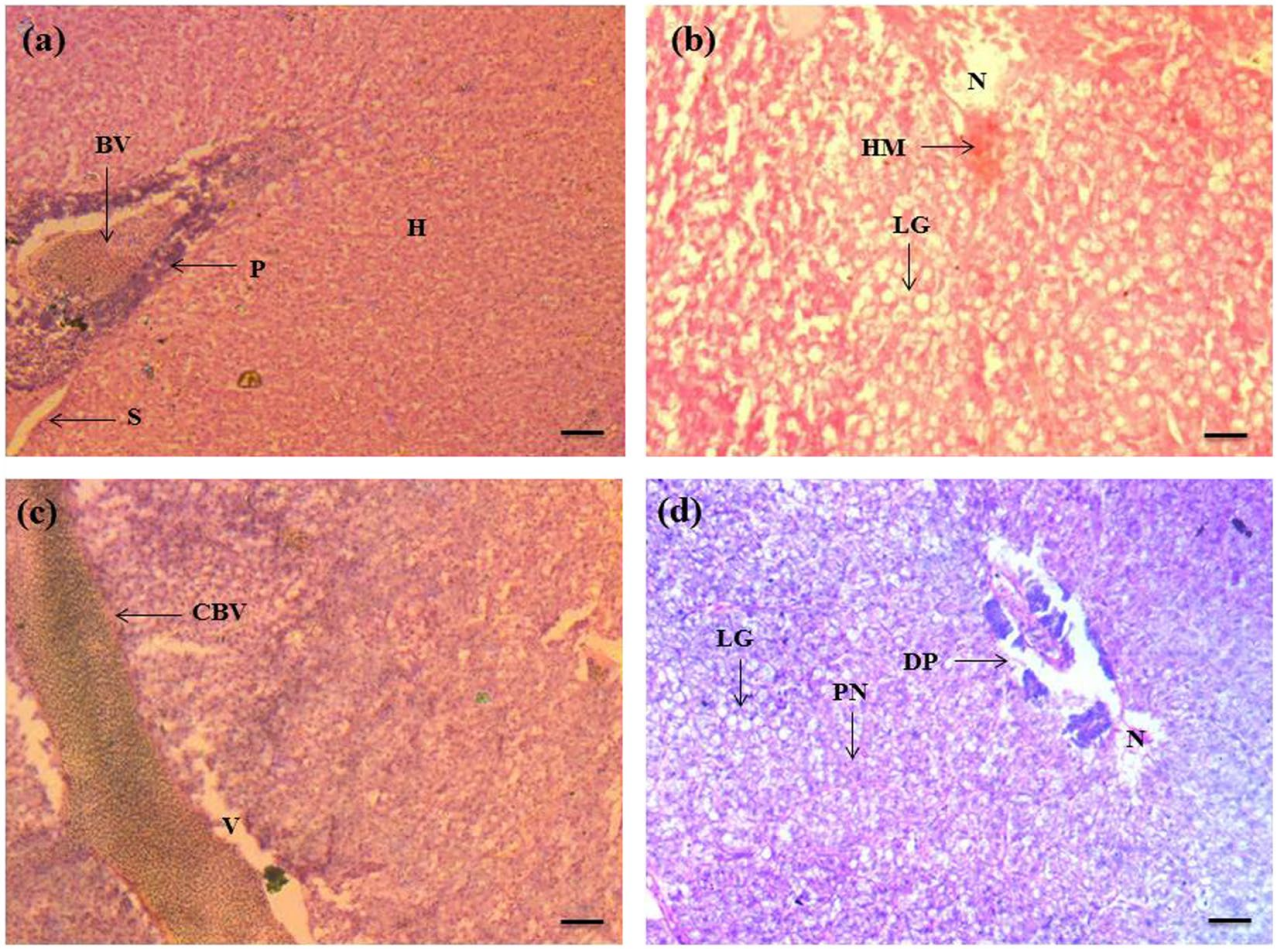
Histopathology of liver of *C. punctatus* inhabiting in reference and contaminated water. (**a**) Reference liver; (**b**, **c** and **d**) Liver of exposed fish; S (sinusoid), BV (blood vessel), P (pancreatic tissue), E (erythrocyte), H (hepatocyte), N (necrosis), V (vacuolization), PN (pyknotic nuclei), DP (degeneration of pancreatic tissue), HM (haemorrhage), CBV (congestion of blood vessel), LG (lipid granules). Scale bar = 20 µm.

**Figure 5:**
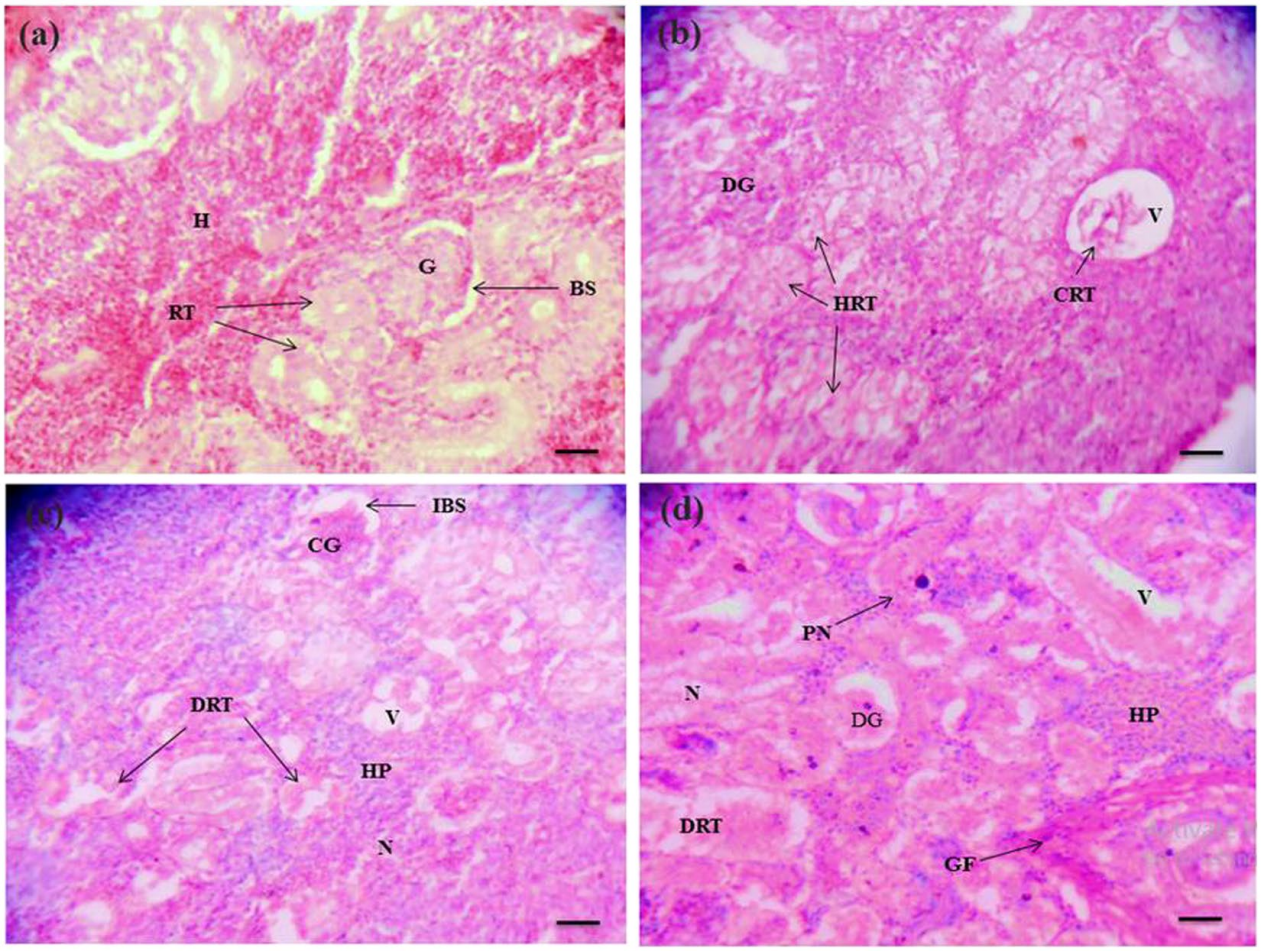
Histopathology of kidney of *C. punctatus* inhabiting in reference and contaminated water. (**a**) Reference kidney; (**b**, **c** and **d**) Kidney of exposed fish; G (glomeruli), BS (bowman's space), RT (renal tubule), H (haemopoietic tissue), IBS (increased bowmans space), DG (damaged glomeruli), DRT (damaged renal tubule), N (necrosis), HRT (hypertrophied renal tubule), V (vacuolation), HP (hyperplasia), CRT (constricted renal tubule), CG (constricted glomeruli), GF (granuloma formation) and PN (pyknotic nuclei). Scale bar = 20 µm.

The histological alterations observed in the gill, liver and kidney of *C*. *punctatus* are shown in Figs 3, 4 and 5 respectively. Histopathological studies of target organs along with the studies of oxidative stress and DNA damage would give the complete picture of heavy metal hazards and their overall toxic potential in aquatic animals. The gill of reference fish showed normal anatomy of secondary lamellae. The structure of heavy metal exposed fish varied from that of the reference. Exposure to waste water ingredients resulted in increased incidence of oedema and hyperplasia in the lamellae, lamellar fusion, gill bridging, epithelial lifting, and necrosis. Gills are the main respiratory organ in fish and are covered by thin epithelium which is the site of exchange of gases, regulation of ionic and acid-base balance and nitrogenous waste excretion^41^. In the current study epithelium was found to be degenerated in the exposed fishes and got separated from the lamellar tissue. The toxic exposure also induced hyperplasia, gill bridging, lamellar fusion in order to increase the distance for diffusion across the cells to reach the bloodstream. This has also been demonstrated by other investigators^29, 34, 42, 43^. These alterations are interpreted as the defense response of fish against heavy metals. Liver of fish is the main metabolic organ where detoxification occurs. Hence, it could also have more chances of degeneration. Liver of the reference fish showed normal hepatocytes with pancrea (hepatopancrea) whereas in the exposed liver, lipid granules, vacuolation, hemorrhage, pyknotic nuclei, congestion of blood vessel and damaged pancrea were prominent. These histological changes are associated with the response of hepatocytes to toxicants and excessive xenobiotic metabolism in liver tissue^44^. Similar observations on histological responses to heavy metal pollution have been reported in the liver of other fish species^37, 45^. Kidney sections of the reference group in Fig. 5(a) showed a normal structure such as glomeruli, bowman’s space with uniform renal tubules and the interstices of the tubules contain hematopoietic tissue. However, increased bowmans space, damaged glomeruli and renal tubule, necrosis, hypertrophied renal tubule, large vacuolation, hyperplasia, constricted renal tubule and glomeruli and granuloma formation in chronologically exposed fish renal tissue (Fig. 5b–d) were the major hallmarks. Necrosis, epithelial tubuli contraction, glomerular injury, reduction of renal hematopoietic system, tissue damage, glomerular constriction and proliferation of connective tissue related with metals contaminated water have also been documented in other reports ^46–48^. Therefore, the histopathological damage may affect the normal physiological functions of the organs.

Heavy metals (Cr, Mn, Fe, Co, Ni, Cu and Zn) contaminated waste water can induce alterations in physiology of *C*. *punctatus*. They are likely to be responsible for oxidative stress, genotoxicity and certain histopathological lesions in gill, liver and kidney of this fresh water fish. *C*. *punctatus* is a suitable bioindicator to monitor water quality in the waste water reservoir.

## Methods

### Ethical statement for animal experimentation

Animal experimentation were permitted by Ministry of Environment and Forests, Government of India under registration no. 714/02/a/CPCSEA issued by Committee for the Purpose of Control and Supervision of Experiments on Animals (CPCSEA) and approved by the institutional ethical committee of Department of Biochemistry, Aligarh Muslim University, Aligarh, India. It has also been confirmed that all methods were performed in accordance with the relevant guidelines and regulations.

### Experimental site, sample collection and analysis

The current study focuses on the Panethi reservoir (27.517° N and 78.175° E), district Aligarh, which receives waste water from multiple sources such as ice factory, mechanical workshops, dairy factory and domestic wastes.

Sampling of both water and fishes were done during April 2016. Water samples were collected in acid rinsed glass bottles from both the Panethi (test) and Sumera (reference) reservoirs. Heavy metals were estimated with the help of atomic absorption spectrophotometer (AAS) as per standard protocols of APHA^49^. Bioavailability was calculated as per equation of Kaviraj and Das^50^.

Live samples of exposed and reference *Channa punctatus* (n = 26; average length 14.32 ± 0.70 cm; average weight 69.6 ± 0.45 g and n = 22, average length 13.53 ± 0.82 cm; average weight 89.25 ± 1.03 g respectively) were collected and brought to the laboratory. Condition factor (K) and hepatosomatic index (HSI%) were calculated according to the equation of Fulton^51^ and Bervoets *et al*.^52^ respectively. Both fishes were euthanized for gill, liver and kidney removal and immediately after dissection they were carefully washed with phosphate buffer. These tissue samples were immediately processed for heavy metal estimation as per standard methods of APHA^49^. For instrument calibration, working standards were prepared by diluting the stock standards (1000 ppm) supplied by Wako Pure Chemical Industry Ltd., Japan. Analytical blanks were also used. The applied analytical procedure accuracy was tested using the certified reference material Dorm-2 (dogfish muscle, National Research Council, Canada) for investigated metals. Replicate analyses of the reference materials showed good accuracy, with recovery rates for Cu 99%, Zn 99%, Fe 99.2%, Ni 98.5%, Cr 98%, Mn 97.7 and Co 97%. Gill, liver and kidney were also used for oxidative stress biomarkers, comet assay and histopathology.

### Glucose, protein and lipid profile of serum

Blood was collected from live fishes through cardiac puncture, kept it stand for some time and thereafter, centrifuged at 3500 rpm for 10 min to obtain serum. Glucose levels were estimated using the kit (Accurex Biomedical Pvt. Ltd., India) and absorbance was read at 505 nm.

Total protein was determined as per standard method of Bradford^53^ taking BSA as a standard. Albumin content was also measured using the kit (Siemens Ltd., Gujarat, India) and absorbance was read against blank at 628 nm. Globulin content was obtained after subtracting albumin from total protein content. Albumin to Globulin (A:G) ratio was also calculated.

Serum total lipid was quantitated using the diagnostic Kit (3830 Valley Centre Dr. San Diego, CA). Total cholesterol was estimated using the cholesterol diagnostic kit (Transasia Bio-Medicals, India) and high density lipoprotein (HDL) cholesterol using kit HDL-C (Siemens Ltd., Gujarat, India). Triglyceride was determined by using the Siemens kit (Siemens Ltd., Gujarat, India). Phospholipids were calculated by the method previously described in Javed and Usmani^6^.

$${\rm{Phospholipid}}={\rm{Cholesterol}}\times 0.73+90$$

Serum very low density lipoprotein (VLDL) and low density lipoprotein (LDL) were calculated according to Friedewald’s equation^54^, as follows:

$${\rm{VLDL}}={\rm{Triglycerides}}/5$$

$${\rm{LDL}}={\rm{Total\; Cholesterol}}\mbox{--}{\rm{Triglycerides}}/5-{\rm{HDL}}$$

### Oxidative stress markers

Homogenates of gill, liver and kidney were prepared in 0.1 M phosphate buffer at pH 7.4.

Superoxide dismutase (SOD) activity was assayed by auto-oxidation of pyrogallol as per the standard protocols of Marklund and Marklund^55^. The reaction mixture contains 2.85 ml of tris-succinate buffer (0.05 M, pH 8.2) and 50 µL of sample. Reaction was started by addition 100 µL of pyrogallol (8 mM) to the reaction mixture and the change in absorbance was measured at 412 nm at an interval of 30 s for 3 min. The specific activity of SOD is expressed as Units mg^−1^ protein. One enzyme unit of SOD is defined as the amounts which cause 50% inhibition of pyrogallolauto-oxidation in a total volume of 3 ml.

Catalase (CAT) was assayed as per the protocols of Aebi^56^ with minor modifications. The reaction mixture containing 50 µL of sample in 1.95 mL of potassium phosphate buffer (50 mM, pH 7.0). After addition of 1 ml of H_2_O_2_ the reaction mixture was monitored at 240 nm for 3 min at an interval of 30 s.

The activity of glutathione S transferase (GST) was measured by the method of Habig *et al*.^57^. The assay mixture contained 2.8 mL of sodium phosphate buffer (0.1 M, pH 6.5), 0.1 ml reduced glutathione and 50 µl sample. GST activity was monitored at 340 nm for 3 min at an interval of 1 min by the addition 50 µl of 1 mM 1-chloro 2, 4 dinitrobenzene (CDNB).

The level of reduced glutathione (GSH) was estimated by method of Jollow *et al*.^58^ with minor modifications. Equal amount of homogenate sample and sulphosalicylic acid were mixed and incubated at 4 °C for 1 h and centrifugation at 12000 rpm for 15 min. The supernatant (0.2 ml) was mixed with 2.6 ml of potassium phosphate buffer (0.1 M, pH 7.4). The reaction was initiated by the addition of 0.2 ml 5, 5′-dithiobis-2-nitrobenzoic acid (DTNB) and the absorbance was monitored at 412 nm.

The extent of lipid peroxidation (LPO) was measured by the standard protocols of Buege and Aust^59^ involving the measurement of total malondialdehyde (MDA) which is the major product of lipid peroxidation. The reaction mixture contains 0.5 ml homogenate sample, 0.5 ml TBA (0.67%) and 0.5 ml TCA (3%). Total reaction mixture was kept in boiling water bath for 20 min, centrifuged at 4000 rpm, 4 °C for 10 min and sample was read at 530 nm.

### Genotoxicity (Comet Assay)

Immediately after dissection, small sections of gills, liver and kidney tissues were transferred into RPMI medium and cellular dissociation was done based on Cavalcante *et al*.^60^. The comet assay was then performed by electrophoresis under alkaline conditions following the protocol of Singh *et al*.^61^, with minor modifications. Cells were embedded in an LMPA sandwich on frosted slides. To remove cellular proteins, slides were submerged in cold lysis buffer and stored at 4 °C for 1 hr in dark. The slides were then allowed to DNA unwinding in alkaline electrophoretic running buffer. Then, electrophoresis was conducted at 0.74 V/cm and 300 mA for 40 min. After electrophoresis, the slides were neutralized and stained with ethidium bromide. The slides were visualized and scored by using Olympus fluorescent microscope (CX41) integrated CC camera with an image analysis system (Komet 5.5, Kinetic imaging, Liverpool, U.K.). The comet images of 50 cells (25 from each replicate slide) for each sample were scored at a magnification of 100× . Comet tail-length (migration of DNA in µm from its nucleus) was chosen as the parameter to assess the nuclear DNA damage.

### Histopathology

For histopathological studies, gills, liver and kidney tissues of exposed and reference fishes were dissected out and fixed in Bouin’s fluid. They were processed for paraffin wax embedding, cut into 4 µm thick sections, and stained with haematoxylin and eosin (H & E) exactly as described by Humason^62^. The slides were examined using a light microscope (Leica DM 2500).

### Statistical analysis

All values were given as mean ± standard error of mean (SEM). Statistical differences among the means of reference and the exposed were determined using Student’s t test using SPSS (version 17) and Duncan’s Multiple Range Test (DMRT)^63^. Levels of significance were established at p < 0.05 and p < 0.01. Assumptions of normality (Shapiro–Wilk test) and homogeneity (Levene’s test) of data were verified^64^.

## Electronic supplementary material

supplementary tables
