# Publisher Correction: Multiple biomarker responses (serum biochemistry, oxidative stress, genotoxicity and histopathology) in *Channa punctatus* exposed to heavy metal loaded waste water

**DOI:** 10.1038/s41598-018-35445-w

**Published:** 2018-11-23

**Authors:** Mehjbeen Javed, Md. Irshad Ahmad, Nazura Usmani, Masood Ahmad

**Affiliations:** 1 Aquatic Toxicology Research Laboratory, Department of Zoology, Aligarh Muslim University, Aligarh, 202002 Uttar Pradesh India; 2 Department of Biochemistry, Faculty of Life Sciences, Aligarh Muslim University, Aligarh, 202002 Uttar Pradesh India

Correction to: *Scientific Reports* 10.1038/s41598-017-01749-6, published online 10 May 2017

In the original version of this Article, Nazura Usmani was incorrectly affiliated with ‘Department of Biochemistry, Faculty of Life Sciences, Aligarh Muslim University, Aligarh, 202002, Uttar Pradesh, India’. The correct affiliation is listed below:

Aquatic Toxicology Research Laboratory, Department of Zoology, Aligarh Muslim University, Aligarh, 202002, Uttar Pradesh, India.

This has now been corrected in the HTML and PDF versions of this Article.

