# Supplementary material for: Multiple biomarker responses (serum biochemistry, oxidative stress, genotoxicity and histopathology) in Channa punctatus exposed to heavy metal loaded waste water
Source: Sci Rep. 2017 May 10;7:1675. doi: 10.1038/s41598-017-01749-6 (PMC5431882; doi:10.1038/s41598-017-01749-6)
Supplement: Supplementary file 1 — supplementary tables [file 41598_2017_1749_MOESM1_ESM.pdf]

Supplementary Material

Multiple biomarker responses (serum biochemistry, oxidative stress, genotoxicity and histopathology) in *Channa punctatus* exposed to heavy metal loaded waste water

Mehjbeen Javed[a,1,*],Md Irshad Ahmad[b,1], NazuraUsmani[a], Masood Ahmad[b]

Supplementary Table S1

Heavy metals	Panethi reservoir	*Sumera reservoir	**Indian (standards)	WHO (guidelines)
Cr (mgL^{-1})	0.08±0.01 (28%)	BDL	0.05	0.05
Mn (mgL^{-1})	2.5±0.05 (77%)	BDL	0.5	0.5
Fe (mgL^{-1})	9±1.66 (97%)	0.24±0.001 (7.7%)	0.3	-
Co (mgL^{-1})	0.26±0.01 (11%)	BDL	-	-
Ni (mgL^{-1})	0.1±0.01 (22.5%)	BDL	-	0.02
Cu (mgL^{-1})	0.08±0.005 (47.6%)	0.10±0.001 (5.3%)	1.5	2
Zn (mgL^{-1})	0.54±0.01 (88.8%)	BDL	15	3

Heavy metal concentrations and their bioavailability in exposed and reference water and their permissible limits; Values are given as mean ± SEM; Samples are replicates of 4; BDL = Below detection limit; WHO guidelines taken from United Nations Environment Programme Global Environment Monitoring System (GEMS)/ Water Programme) (2006); *Reference site; **Drinking water quality guidelines in India taken from IS 2296: 1992; Blank cells indicate that no standards have yet been set; Values in parenthesis indicates bioavailability.

Supplementary Table S2

Heavy metals	Gill	Liver	Kidney
**Cr**	$^{a}76\pm1.40_{e}$	$^{b}23\pm1.32_{e}$	$^{b}10\pm0.01_{e}$
**Mn**	$1369.5\pm2.8_{c}$	BDL	BDL
**Fe**	$^{a}17619\pm4.5_{a}$	$^{b}1501\pm5.2_{a}$	$^{c}3554\pm3.39_{a}$
**Co**	BDL	BDL	BDL
**Ni**	$^{a}31.59\pm1.32_{f}$	$^{b}27\pm1.3_{d}$	$15\pm0.74_{d}$
**Cu**	$^{c}132.8\pm2.38_{d}$	$^{a}165.3\pm2.6_{c}$	$^{b}149.5\pm1.1_{c}$
**Zn**	$^{a}1845\pm3.32_{b}$	$^{c}875\pm4.2_{b}$	$^{b}1221\pm2.3_{b}$
**Reference *C. punctatus***			
**Fe**	$^{a}12.5\pm1.01_{a}$	$^{b}7.6\pm0.41_{a}$	$^{c}3\pm0.20_{a}$
**Cu**	$^{a}8.9\pm0.40_{b}$	$^{b}3.01\pm0.4_{b}$	$^{c}1.98\pm0.06_{b}$

Accumulation of heavy metals in tissues of *Channa punctatus* collected from of Panethi and Sumera reservoir; All values are given as mean ± SEM, (n= 12), BDL= Below detection limit; DMRT was used to test the significant difference among means. Means with similar letters in a column and row are statistically insignificant at $p < 0.01$; Superscripts indicates the accumulation of a heavy metal ($mgkg^{-1}$.dry weight) in various tissues of the fish; Subscripts indicates the accumulation of different heavy metals ($mgkg^{-1}$.dry weight) in a particular tissue of the fish
